# Supplementary material for: Bayesian refinement of protein structures and ensembles against SAXS data using molecular dynamics
Source: PLoS Comput Biol. 2017 Oct 18;13(10):e1005800. doi: 10.1371/journal.pcbi.1005800 (PMC5662244; doi:10.1371/journal.pcbi.1005800)
Supplement: S1 Archive — (BZ2) [file pcbi.1005800.s011.tar.bz2 › gromacs_4.62_bayesian_saxs_refi/share/html/online/g_wham.html]

g\_wham


| g\_wham Main Table of Contents |

**VERSION 4.6  
Sat 19 Jan 2013**

---

### Description

This is an analysis program that implements the Weighted
Histogram Analysis Method (WHAM). It is intended to analyze
output files generated by umbrella sampling simulations to
compute a potential of mean force (PMF).

At present, three input modes are supported.  
\* With option -it, the user provides a file which contains the
file names of the umbrella simulation run-input files (.tpr files),
AND, with option -ix, a file which contains file names of
the pullx mdrun output files. The .tpr and pullx files must
be in corresponding order, i.e. the first .tpr created the
first pullx, etc.  
\* Same as the previous input mode, except that the the user
provides the pull force output file names (pullf.xvg) with option -if.
From the pull force the position in the umbrella potential is
computed. This does not work with tabulated umbrella potentials.  
\* With option -ip, the user provides file names of (gzipped) .pdo files, i.e.
the GROMACS 3.3 umbrella output files. If you have some unusual reaction coordinate you may also generate your own .pdo files and
feed them with the -ip option into to g\_wham. The .pdo file header
must be similar to the following:

# UMBRELLA 3.0  
# Component selection: 0 0 1  
# nSkip 1  
# Ref. Group 'TestAtom'  
# Nr. of pull groups 2  
# Group 1 'GR1' Umb. Pos. 5.0 Umb. Cons. 1000.0  
# Group 2 'GR2' Umb. Pos. 2.0 Umb. Cons. 500.0  
#####

The number of pull groups, umbrella positions, force constants, and names
may (of course) differ. Following the header, a time column and
a data column for each pull group follows (i.e. the displacement
with respect to the umbrella center). Up to four pull groups are possible
per .pdo file at present.

By default, the output files are  
-o PMF output file  
-hist Histograms output file  
Always check whether the histograms sufficiently overlap.

The umbrella potential is assumed to be harmonic and the force constants are
read from the .tpr or .pdo files. If a non-harmonic umbrella force was applied
a tabulated potential can be provided with -tab.

WHAM OPTIONS  
------------  
-bins Number of bins used in analysis  
-temp Temperature in the simulations  
-tol Stop iteration if profile (probability) changed less than tolerance  
-auto Automatic determination of boundaries  
-min,-max Boundaries of the profile   
The data points that are used to compute the profile
can be restricted with options -b, -e, and -dt.
Adjust -b to ensure sufficient equilibration in each
umbrella window.

With -log (default) the profile is written in energy units, otherwise
(with -nolog) as probability. The unit can be specified with -unit.
With energy output, the energy in the first bin is defined to be zero.
If you want the free energy at a different
position to be zero, set -zprof0 (useful with bootstrapping, see below).

For cyclic or periodic reaction coordinates (dihedral angle, channel PMF
without osmotic gradient), the option -cycl is useful. g\_wham will make use of the
periodicity of the system and generate a periodic PMF. The first and the last bin of the
reaction coordinate will assumed be be neighbors.

Option -sym symmetrizes the profile around z=0 before output,
which may be useful for, e.g. membranes.

AUTOCORRELATIONS  
----------------  
With -ac, g\_wham estimates the integrated autocorrelation
time (IACT) τ for each umbrella window and weights the respective
window with 1/[1+2\*τ/dt]. The IACTs are written
to the file defined with -oiact. In verbose mode, all
autocorrelation functions (ACFs) are written to hist\_autocorr.xvg.
Because the IACTs can be severely underestimated in case of limited
sampling, option -acsig allows one to smooth the IACTs along the
reaction coordinate with a Gaussian (σ provided with -acsig,
see output in iact.xvg). Note that the IACTs are estimated by simple
integration of the ACFs while the ACFs are larger 0.05.
If you prefer to compute the IACTs by a more sophisticated (but possibly
less robust) method such as fitting to a double exponential, you can
compute the IACTs with g\_analyze and provide them to g\_wham with the file
iact-in.dat (option -iiact), which should contain one line per
input file (.pdo or pullx/f file) and one column per pull group in the respective file.

ERROR ANALYSIS  
--------------  
Statistical errors may be estimated with bootstrap analysis. Use it with care,
otherwise the statistical error may be substantially underestimated.
More background and examples for the bootstrap technique can be found in
Hub, de Groot and Van der Spoel, JCTC (2010) 6: 3713-3720.  
-nBootstrap defines the number of bootstraps (use, e.g., 100).
Four bootstrapping methods are supported and
selected with -bs-method.  
(1) b-hist Default: complete histograms are considered as independent
data points, and the bootstrap is carried out by assigning random weights to the
histograms ("Bayesian bootstrap"). Note that each point along the reaction coordinate
must be covered by multiple independent histograms (e.g. 10 histograms), otherwise the
statistical error is underestimated.  
(2) hist Complete histograms are considered as independent data points.
For each bootstrap, N histograms are randomly chosen from the N given histograms
(allowing duplication, i.e. sampling with replacement).
To avoid gaps without data along the reaction coordinate blocks of histograms
(-histbs-block) may be defined. In that case, the given histograms are
divided into blocks and only histograms within each block are mixed. Note that
the histograms within each block must be representative for all possible histograms,
otherwise the statistical error is underestimated.  
(3) traj The given histograms are used to generate new random trajectories,
such that the generated data points are distributed according the given histograms
and properly autocorrelated. The autocorrelation time (ACT) for each window must be
known, so use -ac or provide the ACT with -iiact. If the ACT of all
windows are identical (and known), you can also provide them with -bs-tau.
Note that this method may severely underestimate the error in case of limited sampling,
that is if individual histograms do not represent the complete phase space at
the respective positions.  
(4) traj-gauss The same as method traj, but the trajectories are
not bootstrapped from the umbrella histograms but from Gaussians with the average
and width of the umbrella histograms. That method yields similar error estimates
like method traj.

Bootstrapping output:  
-bsres Average profile and standard deviations  
-bsprof All bootstrapping profiles  
With -vbs (verbose bootstrapping), the histograms of each bootstrap are written,
and, with bootstrap method traj, the cumulative distribution functions of
the histograms.

### Files

| option | filename | type | description |
| --- | --- | --- | --- |
| **-ix** | pullx-files.dat | Input, Opt. | Generic data file |
| **-if** | pullf-files.dat | Input, Opt. | Generic data file |
| **-it** | tpr-files.dat | Input, Opt. | Generic data file |
| **-ip** | pdo-files.dat | Input, Opt. | Generic data file |
| **-o** | profile.xvg | Output | xvgr/xmgr file |
| **-hist** | histo.xvg | Output | xvgr/xmgr file |
| **-oiact** | iact.xvg | Output, Opt. | xvgr/xmgr file |
| **-iiact** | iact-in.dat | Input, Opt. | Generic data file |
| **-bsres** | bsResult.xvg | Output, Opt. | xvgr/xmgr file |
| **-bsprof** | bsProfs.xvg | Output, Opt. | xvgr/xmgr file |
| **-tab** | umb-pot.dat | Input, Opt. | Generic data file |

### Other options

| option | type | default | description |
| --- | --- | --- | --- |
| **-[no]h** | bool | no | Print help info and quit || **-[no]version** | bool | no | Print version info and quit || **-nice** | int | 19 | Set the nicelevel || **-xvg** | enum | xmgrace | xvg plot formatting: xmgrace, xmgr or none || **-min** | real | 0 | Minimum coordinate in profile || **-max** | real | 0 | Maximum coordinate in profile || **-[no]auto** | bool | yes | Determine min and max automatically || **-bins** | int | 200 | Number of bins in profile || **-temp** | real | 298 | Temperature || **-tol** | real | 1e-06 | Tolerance || **-[no]v** | bool | no | Verbose mode || **-b** | real | 50 | First time to analyse (ps) || **-e** | real | 1e+20 | Last time to analyse (ps) || **-dt** | real | 0 | Analyse only every dt ps || **-[no]histonly** | bool | no | Write histograms and exit || **-[no]boundsonly** | bool | no | Determine min and max and exit (with -auto) || **-[no]log** | bool | yes | Calculate the log of the profile before printing || **-unit** | enum | kJ | Energy unit in case of log output: kJ, kCal or kT || **-zprof0** | real | 0 | Define profile to 0.0 at this position (with -log) || **-[no]cycl** | bool | no | Create cyclic/periodic profile. Assumes min and max are the same point. || **-[no]sym** | bool | no | Symmetrize profile around z=0 || **-[no]ac** | bool | no | Calculate integrated autocorrelation times and use in wham || **-acsig** | real | 0 | Smooth autocorrelation times along reaction coordinate with Gaussian of this σ || **-ac-trestart** | real | 1 | When computing autocorrelation functions, restart computing every .. (ps) || **-nBootstrap** | int | 0 | nr of bootstraps to estimate statistical uncertainty (e.g., 200) || **-bs-method** | enum | b-hist | Bootstrap method: b-hist, hist, traj or traj-gauss || **-bs-tau** | real | 0 | Autocorrelation time (ACT) assumed for all histograms. Use option -ac if ACT is unknown. || **-bs-seed** | int | -1 | Seed for bootstrapping. (-1 = use time) || **-histbs-block** | int | 8 | When mixing histograms only mix within blocks of -histbs-block. || **-[no]vbs** | bool | no | Verbose bootstrapping. Print the CDFs and a histogram file for each bootstrap. |

---

http://www.gromacs.org  
gromacs@gromacs.org
